# Getting off on the wrong foot? How community groups in Zimbabwe position themselves for partnerships with external agencies in the HIV response

**DOI:** 10.1186/s12992-017-0253-5

**Published:** 2017-06-01

**Authors:** Morten Skovdal, Sitholubuhle Magutshwa-Zitha, Catherine Campbell, Constance Nyamukapa, Simon Gregson

**Affiliations:** 1 Department of Public Health, University of Copenhagen, Øster Farimagsgade 5, 1014 Copenhagen, Denmark; 2 Biomedical Research and Training Institute, Harare, Zimbabwe; 3 Department of Psychological and Behavioural Science, London School of Economics and Political Science, London, UK; 4 School of Public Health, Imperial College London, London, UK

**Keywords:** Community response, HIV, Partnerships, NGOs, CBOs, Zimbabwe

## Abstract

**Background:**

Partnerships are core to global public health responses. The HIV field embraces partnership working, with growing attention given to the benefits of involving community groups in the HIV response. However, little has been done to unpack the social psychological foundation of partnership working between well-resourced organisations and community groups, and how community representations of partnerships and power asymmetries shape the formation of partnerships for global health. We draw on a psychosocial theory of partnerships to examine community group members’ understanding of self and other as they position themselves for partnerships with non-governmental organisations.

**Methods:**

This mixed qualitative methods study was conducted in the Matobo district of Matabeleland South province in Zimbabwe. The study draws on the perspectives of 90 community group members (29 men and 61 women) who participated in a total of 19 individual in-depth interviews and 9 focus group discussions (*n* = 71). The participants represented an array of different community groups and different levels of experience of working with NGOs. Verbatim transcripts were imported into Atlas.Ti for thematic indexing and analysis.

**Results:**

Group members felt they played a central role in the HIV response. Accepting there is a limit to what they can do in isolation, they actively sought to position themselves as potential partners for NGOs. Partnerships with NGOs were said to enable community groups to respond more effectively as well as boost their motivation and morale. However, group members were also acutely aware of how they should act and perform if they were to qualify for a partnership. They spoke about how they had to adopt various strategies to become attractive partners and ‘supportable’ – including being active and obedient.

**Conclusions:**

Many community groups in Zimbabwe recognise their role in the HIV response and actively navigate representational systems of self and other to showcase themselves as capable actors. While this commitment is admirable, the dynamics that govern this process reflect knowledge encounters and power asymmetries that are conditioned by the aid architecture, undermining aspiring efforts for more equitable partnerships from the get-go.

## Background

The global HIV response, like so many international health and development responses, relies on productive partnerships between multiple stakeholders. Community groups have always played a critical role in the HIV response, whether it is through ‘behind the scene’ support, or induced more formally through partnerships with aid and development agencies [1]. Partnerships between local community structures and well-resourced external agencies are generally presumed critical for sustainable and successful global health responses. Community structures are generally seen as ‘close to the ground’ and able to reach ‘those most in need’ [2]. Partnership-working also has the potential to ensure that health interventions build on already existing community processes and structures, create locally appropriate and locally owned responses to health needs, and link community structures to otherwise unobtainable resources, such as technical assistance and funding [3–6].

Treating community structures as indispensible stakeholders in the HIV response is gaining momentum in much HIV programming policy and research. From a policy perspective, the Strategic Investment Framework posits community engagement and actions as ‘critical enablers’ for an effective HIV response [7, 8]. Recent World Health Organisation guidelines stress the importance of engaging communities in the delivery of HIV services. Guidelines on how to monitor national and international health sector responses to HIV see community groups as part and parcel of the health sector [9]. The consolidated guidelines on the use of antiretroviral drugs for treating and preventing HIV infection stipulate the need for community-centred and community led health care approaches [10]. The need to involve community structures in the HIV response is also recognised by the UNAIDS in their Fast-Track targets. They predict that community structures will play an ever increasing role in the HIV response and estimate that the proportion of global resource needs for community engagement activities will quadruple from 1% in 2014 to 4% in 2030 [11]. Commentators, however, note that we are long way from translating these policy commitments into action, truly recognising community structures as assets, and call for a paradigm shift in how we approach and involve local community structures [12].

Research indicates that community structures have much to offer the HIV response. We have in previous work shown that community group membership can have a positive effect against HIV infection for women [13, 14] and encourage faster uptake of HIV services [15]. A major World Bank report, evaluating 15 studies carried out in 8 countries, found community structures to make a critical contribution to the HIV response by helping to mobilise local resources, improve knowledge and behaviour, increase use of HIV services, instigate social change for improved health and reduce HIV incidence [16]. Randomised trials are beginning to emerge, pointing to the benefits of mobilising and involving community structures to ‘translate’ health services, often rooted in behavioural and biomedical interventions, into services that are accessible, relevant and responsive to local needs – enhancing both reach and impact. Project Accept, for example, implemented in 48 communities across Zimbabwe, South Africa, Tanzania and Thailand, found community mobilisation activities, such as community working groups, community ‘recruiters’, post-test psychosocial support and mobile out-reach testing services to contribute to a 25% increase in HIV testing and a reduction in HIV incidence of 13·9% [17]. Community structures can also be mobilised to challenge health-damaging norms and behaviours. SASA!, a trial in Uganda, has documented significantly lower levels of social acceptability of intimate partner violence in intervention sites where community structures were engaged through local community activists to critically reflect on gender norms and power asymmetries [18, 19]. Promising as this is, fine-grained analyses of partnership-working in real-life settings indicate that power asymmetries between ‘local’ and ‘global’ actors and knowledge systems, may undermine the success potential of such initiatives [20, 21].

Looking at NGO-CBO partnerships for HIV prevention amongst sex workers in India, Cornish and colleagues [22] found NGOs, despite their commitment to empower and engage local community structures, to undermine this potential by prescribing global management standards. Community organisations, in order to access resources, had to conform to certain funding practices, professionalising them in a way that distanced them from the very localism that attracted them funding in the first place. Similar observations have been made by Aveling [23], who in a study of aid-chain funding in the HIV response in Cambodia, found international NGOs to position local NGOs and community structures as recipients of aid and to prioritise capacity building for fund management. Aveling argues that while this provides local actors with important material and social capital in the HIV response, such representations and professionalising activities simultaneously perpetuate the inequalities that exist between international NGOs and local actors, undermining community leadership and recognition of local strengths. Nair and Campbell [24], reflecting on their experiences as facilitators of partnership-formations in South Africa, note both a misrecognition of local strengths (the capacity of community health volunteers to address challenges of the HIV epidemic), and the limited capacity of external change agents (who in turn are steered by their donors) to work productively with local actors in support of a community response, obstructing goals of local ownership and equal stakeholder involvement. They go on to argue that rather than focusing on the capacity needs of local actors, there is an equal need to strengthen the skills and competencies of HIV service providers to work with community structures.

These examples illustrate the challenge of putting partnership-working into practice. To build the competence of HIV service providers, and to address the challenges outlined above, we need to scrutinize the social psychological foundation of partnership-working in the HIV response, a hitherto under-explored area of study: How do community groups position themselves for partnerships with external agencies? How do representations of self and other shape the foundation of aspiring partnerships? How do power-asymmetries, reflecting the aid architecture, manifest themselves in community groups’ strategic positioning? These are some of the questions that we explore in this paper, as we seek to unpack the foundation of partnership-working between community groups and non-governmental organisations (NGOs) in the context of HIV in Zimbabwe.

### Conceptual framework

To help us do this, we draw on a social psychological theory that provides a framework for analysing the dynamics of partnership-working. Drawing on learning from partnership-working in Cambodia and Brazil, Aveling and Jovchelovitch [25] stipulate that to understand and analyse partnership-working, we need to recognise the interplay between three dimensions: i) representations of self and other; ii) styles of communication; and iii) representational projects. *Representations of self and other* refer to the shared stock of values, ideas and practices that establish an order, as well as codes for social exchanges, through which partners are able to orient and position themselves. This encourages us to investigate how community structures describe themselves and their role in the HIV response, particularly in relation to other stakeholders, and the impact of these representations on their positioning. In *styles of communication*, Aveling and Jovchelovitch [25] draw attention to the communicative strategies employed by partners to demonstrate their power and positioning. Given their focus on partnership-working, Aveling and Jovchelovitch emphasise the patterns of communication that take place while working together. As we are interested in the prospects of partnership-working, at a stage where community groups and NGOs may not yet have exchanged words, we will focus on how community structures communicate their position to NGOs through action. This allows us to explore how community groups, through communicative action [26], mobilise, coordinate, and position themselves for partnerships, based on a collective understanding of their role in the HIV response. *Representational projects* refer to the teleological and future-building aims of partners [27]. These dimensions encourage us to ‘zoom in’ on how community groups see themselves through the eyes of external change agents, measuring and positioning themselves in relation to external expectations. We use the framework to disentangle the role of past experience and knowledge of each other in shaping future partnership-working.

## Methods

We draw on data from a qualitative study that sought to explore community responses to HIV in southern Zimbabwe. The study forms part of an on-going research project with ethical approval from the Medical Research Council of Zimbabwe (A/681) and Imperial College London (ICREC_9_3_13). Informed and written consent were gathered from all research participants with the assurance that their identities would not be revealed. Pseudonyms have therefore been used throughout.

### Study location and participants

The study took place in the Matobo District of Matabeleland South Province, Zimbabwe. The district has a population of 110,000 people and a HIV prevalence rate of 22.3% [28]. The northern part of the district is characterised by an arid landscape, making cattle and goat keeping the primary source of income for residents. The south of the District offers greater opportunities for small-scale and subsistence farming. The District borders South Africa to the south and Botswana to the west, whose industry, cash crop farming and mining companies attract a significant number of Matobo men who are looking for work. At the time of the study, Matobo District had 19 international (e.g., Save the Children, Mildmay, Red Cross and World Vision) and local (e.g., Maranatha, Sikhethimpilo and Jairos Jiri) organisations present that were collaborating with community members and groups. In addition to HIV work, many of these organisations also attended to the water and food shortages experienced by the people of Matobo.

Researchers from the Biomedical Research and Training Institute invited 90 community group members to participate in this study. They were identified in consultation with community guides and a representative from the District AIDS Action Committee. The community group members were sampled to represent an array of different community groups and included members of a church group, an AIDS support group, a burial society, a rotating credit society, a women’s group, a sports club, a youth group, a co-operative and a farmer’s group (see Table 1). All participants were over the age of 18.

**Table 1:** Community groups in Matobo District, Matabeleland South, southern Zimbabwe

Group	Description
Church group	Members from the same congregation meet outside of regular church worship times. Engage in Bible study, discussing marital issues, and community outreach, particularly helping families in need (such as those with sick members or orphaned children)
AIDS support group	Loose term to apply to variety of groups including Post HIV test clubs (mostly PLWHA), HIV/ART support groups often organized by clinics, youth groups, peer education groups, home based care groups (members go house to house helping families with sick relatives - doing chores, bathing the sick, sometimes collecting pills from clinic, etc.)
Burial society	Members contribute small sums of money to a central fund to cover basic funeral expenses for themselves and other members. Members commit to organizing proper burials for one another and often sing at funerals. Generally meet monthly.
Savings and lending group	Members contribute to a central fund and when they reach a certain amount the money is shared for income generating projects such as buying seeds. Members borrow at the same interest rate and loans can be made to non-members at a higher rate.
Women’s group	Often linked to Government women’s empowerment initiatives. Supported by Government income generating grants.
Sports club	Male dominated. Organize tournaments against other regions. Primarily soccer.
Youth group	Often organized by political parties or teachers, these seek to develop leadership skills and provide recreation for youth (often into 20s – ‘end of youth’ often determined by marriage)
Co-operative	Group members come together to set up an income generating project, co-owned and run by members. The groups sometimes get assistance from NGOs to expand their work.
Farmer’s group	Farmers, both male and female, meet monthly to plant crops, discuss weather patterns and new technologies, share labour and access NGO assistance (e.g. for farming implements or water irrigation)

### Data collection and analysis

We interviewed group members through a mix of individual semi-structured interviews (IDIs) and focus group discussions (FGDs) (see Table 2). Interviews were conducted in the group’s regular meeting place by trained and experienced researchers who carried out the interviews in the local Ndebele language. Interviews were digitally recorded. The individual semi-structured interviews lasted an average of 90 min, whilst the focus group discussions lasted approximately 120 min. To compensate them for their time and expenses, we provided each participant with two bars of soap, lunch and reimbursement of transport costs.

**Table 2:** Participant characteristics

Type of informants	IDIs	FGDs	Total
AIDS support group members	2 women, 1 man	1 (8 women and 1 man)	12
Burial society group members	2 women	1 (4 women and 2 men)	8
Church group members	1 woman, 1 man	1 (11 women)	13
Cooperative members	1 woman	1 (7 women and 1 man)	9
Farmers group members	2 women, 1 man	1 (4 women and 5 men)	12
Savings and lending group members	3 women	1 (5 women and 1 man)	9
Soccer club members	3 men	1 (8 men)	11
Women’s group members	2 women	1 (4 women)	6
Youth group members	-	1 (5 women and 5 men)	10
Total no. of participants	19	9 FGDs (71 participants)	90

The interviews and FGDs were conducted using a topic guide designed to explore community group members’ perceptions of their role in the HIV response. Participants were first asked to reflect on the impact of HIV on community life and community strategies to support people affected by HIV. This was followed by questions about community strengths, resources and obstacles in their support for people living with HIV as well as the role of networking and partnerships in the community response to HIV. It was the thriving discussions emerging from this latter cluster of questions that gave rise to this article.

Audio recordings were transcribed and translated from Ndebele into English and imported into Atlas.Ti7, a computer-assisted qualitative analysis software. Transcripts were read carefully before the coding process started. A total of 96 codes, encompassing 907 text segments, or quotations, emerged inductively from this process – detailing community responses to HIV. This paper does not seek to report on the entire data set, but focuses on the 38 codes, encompassing 397 text segments (44% of all data) that speak to community strengths, resources and obstacles as well as networking and partnerships. Following the thematic network analysis steps proposed by Attride-Stirling [29], a further layer of analysis, drawing on our conceptual framework was conducted. This resulted in codes being analytically grouped together into basic themes and more interpretative organising themes (see Table 3). We will now discuss each of the basic themes emerging from our analysis under headings reflecting our organising themes.

**Table 3:** Thematic network analysis: from basic themes to organising themes

Basic Themes	Organizing Themes
Community groups support PLWHA and orphaned and vulnerable children	Representations of self: “It is very good for us to work together”
Community group members support each other	
Community groups cannot respond effectively to HIV on their own	
Collaboration is necessary, provides credibility and motivation	
NGOs set the priorities	Representations of other: “they [NGOs] have the final say, and we just do what they say”
NGOs expect obedience	
NGOs may not approach the community ‘the right way’	
Active community groups more likely to be selected by NGOs as partners	Communicative strategy: “they [NGOs] should not be met by lazy people”
Community groups distance themselves from ‘laziness’	

## Results

### Representations of self: “it is very good for us to work together”

The study participants proudly discussed their role in the HIV response. This was particularly noticeable amongst members from community groups that have it within their mandate to mitigate the impact of HIV. Members from the HIV/AIDS support group, for example, spoke about their role in reducing HIV transmission, and to get people tested for HIV, through encouragement and being open about their positive status. This openness is illustrated by Gertrude, one of the oldest members of the HIV/AIDS support group, who reflected on their role in creating HIV awareness.

“We talk about AIDS and our HIV status in every gathering so people are getting used to it and are starting to realise that we should learn and accept that HIV and AIDS is here amongst us.” Gertrude, age 62, member of a HIV/AIDS support group

Also members of community groups that build on an ethic of care, such as the women’s group and the church group, spoke about their role in the HIV response. They saw it as within their remit to offer care and support for orphaned and vulnerable children and people living with HIV, as exemplified by Nenezelani, a Church group member.

“During the week, we go and visit the sick in hospital; we go and visit and pray for those who will have been admitted in hospital.” Nenezelani, Church group member in a FGD (MA-CH-FGD-1)

Not all the community groups were established with a specific purpose to respond to the HIV epidemic. Nonetheless, most of the groups provided support to people affected by HIV in indirect ways. For example the farmers’ association provided members with the skills to farm as well as access to farming implements from NGOs, opening up opportunities for income generation in a community where poverty has dramatically exacerbated the impacts of AIDS. The burial society provided its members with the insurance of burial support following the bereavement of self or a close family member. However, common across all the FGDs was recognition that participation in community groups provides a safety net for people living in low resource and high HIV prevalence communities.

“I joined the group in 2011, in the group I get to discuss with my friends and we assist one another in a number of ways, and they can tell me what to do when I am faced with some problems and we think that if we work together then we can have our garden and plant vegetables like carrots and other nutritious vegetables so that we can get money.” Octavia, age 25, member of a HIV/AIDS support group

“I joined the Burial because it helps, alone you cannot manage, since we are here in rural areas we do not work, we will be managing the little money that we get to assist others so that when you face a problem they will be able to assist you so that you are able to do something, that is why I joined, realizing that alone I cannot carry the problem in a single day, the money is difficult to come by.” Susan, age 51, member of a burial society

These observations suggest that members of formal community groups represent themselves, and their groups, as key structures in the HIV response. They described how ‘coming together’ in groups can lead to activities that help non-members mitigate the impact of HIV, while simultaneously serve as an important safety net for members. In other words, community group structures provide members with a platform to enact and project their agency in dealing with life’s challenges.

While they saw themselves as capable and important actors in the HIV response, they also recognised that there is a limit to what they, as individual groups, can do on their own. The notion of ‘coming together’ also applied to a group-level, with community groups and local NGOs working together. However, the way the community groups discussed the importance of teaming up with other organisations varied. As demonstrated by Sifelumusa, members of the church group spoke about partnerships as opportunities for mutual learning and shared decision making on what actions to take.

“I think it is very good for us to work together and with other organizations and get different ideas on how they see it and also how we see it and then we bring the ideas together and see what we can do to succeed.” Sifelumusa, female age 30, member of a church group

In the FGD with members from a savings and lending group, the discussion was framed by their past experience of being supported by an NGO. They received a series of capacity building training sessions, ‘professionalising’ their rotating and savings activities. They described how this provided them with the skills and know-how needed to succeed, and empowered them to teach others about how to organise themselves ‘professionally’. This is illustrated by an account from Godfrey, who also highlights how they, as NGO trained ‘professionals’, are staging plays about a so-called ‘laziness’, something we will return to later.

When it was realised that we were “moving” but not in any progressive way, that is when we were trained by ORAP on how to do things as you can see we now have the secretaries that you mentioned earlier. The treasurers were also given training on how to handle the finances and we started to note some improvements. Nowadays we carry out some “consults” every now and then, we are inviting others from different groups and we come together as five or six groups and we make these small contributions which go to one group for the hosting of that gathering. Goods are sold, people eat and drink, and there are stage plays about laziness and prevention and awareness of the disease. Godfrey, male age 63, member of a savings and lending group

Partnering with NGOs provides community groups with credibility, as illustrated by the rotating and savings association, who, as a result of their connection with a credible organisation, can now provide services to other groups. However, there are hierarchies of external change agents, with some providing greater credibility and motivation than others. For instance, Raymond, from a farmers association, described in a FGD how activities initiated by international NGOs are considered more desirable and credible compared to locally driven intiatives.

“The strength of their [international NGOs] support services comes from the perception that locals have of any help that comes from outside the community. It is viewed in very high regard when compared with what can come from within the community. For example, if an announcement was to be made that someone from the UK was in the community to share information on HIV/AIDS, the people would be more excited about the event than if it was the same announcement but this time with Mr Mguni in place of the visitor. It sounds more credible and acceptable if it has the backing of an organisation than when it is initiated by local people” Raymond, male, age 44, member of a farmers association

This section has demonstrated how community group members in this particular context represent themselves as key actors in the HIV response. In recognising their limits, both as individuals, and individual groups, they placed great emphasis on ‘coming together’ and ‘working together’. Working with well-resourced international organisations in the HIV response was considered a key motivator and goal for many of the community groups.

### Representations of other: “they [NGOs] have the final say, and we just do what they say”

NGOs were described as rigid and having very clear ideas about ‘what’ and ‘who’ to support, and as doing very little to involve, let alone consult, community groups in the decision making. This transpired in all of our interviews, and is well summarised by Albert, a member of a soccer club:

“What I have realized is that when an Organisation visits they will state clearly the kind of people they want to participate in their programme” Albert, male age 41, member of a soccer club

Not only were partner NGOs described as prescriptive in their selection of beneficiaries for a project, a number of participants spoke about how NGOs failed to target the most vulnerable members of their community, or were simply too restrictive in their focus.

“Sometimes you find a poor person who is suffering not getting help but someone who is not poor getting help from the organizations, maybe it is us the people who do not communicate well here, but it is painful indeed. You find that someone is really poor, maybe it’s an old woman failing to get anything but some who is a bit better getting something.” Constance, age 51, member of a burial society

“I think the major shortcomings that these people who come from outside have are that their support is not wholesome it is one sided they may only look at providing support for the person who is suffering from AIDS and stop short of extending that support to the people who are living with the person.” Peter, age 69, member of a farmers association.

Building on Peter’s perspective, Elias, also a member of a farmers association, noted that NGO support often consists of ‘pre-packaged solutions’, offering services that do not necessarily match the experienced needs of their intended beneficiaries. He argued for NGOs to engage with people locally to learn about their needs.

“What I have noted to be the weakness of these organisations is that they come to the area with pre-packaged aid solutions which might not be exactly what the intended beneficiaries’ need. Like you can come here and give me salt, but I have salt at home, what I want is tea leaves, you know what I mean? Their support may be great but it is specific and they mean well but maybe a little ‘research’ is needed to find out what the people need before the support is sent to them so that when the support finally reaches the people it is specific and more effective.” Elias, age 44, member of a farmers association

The lack of community engagement in designing and planning interventions was salient in all of the interview data. When communities were approached by NGOs, it was often, as articulated by Julia from a HIV/AIDS support group, merely a matter of informing community members about a programme and inviting groups, willing to adhere to their terms and conditions, to join them as partners:

“When we are working with these big Organisations and they are the ones who will be funding the whole project so they have the final say, and we just do what they say […] When they come to the community they will call us and tell us about the kind of support they have to offer and ask if we want it and if we understand what it is about. So if we say we agree them that is when they will start working with us, they do not force us, they want to know if we want to work with them and agree to their terms, then they start.” Julia, female age 56, member of HIV/AIDS support group

With only few exceptions, the study participants painted an unflattering image of NGOs as authoritarian and prescriptive, requiring a level of obedience from their local community partners. These representations provide context and background to the perceived values, ideas and practices of NGOs that community groups position themselves in relation to, as they strive to be considered attractive and ‘supportable’ to NGOs.

### Communicative strategy: “they [NGOs] should not be met by lazy people”

The section above revealed a social representation that NGOs favour community structures that are active agents. Unsurprisingly, the two communicative strategies that we found community members to adopt – in positioning themselves as ‘supportable’ – pertained to portraying a sense of dutifulness to development. One way of communicating this commitment to NGOs was through the formation of community groups. Tom illustrates this by arguing that only by working together in groups will they be able to attract NGOs to their areas:

“People from Mat South are marginalised when it comes to a lot of things and it is not easy for aid to reach them, so with these kind of clubs we will be able to attract other organisations to assist us in making our area move forward and develop.” Tom, age 56, member of an informal savings society

But establishing groups, as a form of communicative action, was not enough. Another communicative strategy adopted by the community groups related to portraying agency and rejecting, what some participants referred to as ‘laziness’. This is demonstrated by both Simon, a member of a youth group, and John, a member of a savings and lending group.

“For us to work smoothly we cannot be lazy. If they sponsor us with materials to help us progress in our lives, we are supposed to be able to do something with it and not just sit on it. Sitting on the material kills the relationship, because they cannot sponsor a lazy person when they could support people who will be able to use the material to progress in life. They will not be able to support someone who is not supportable.” Simon, age 20, member of a youth group

“When organisations from outside the community come to provide us with services, they should not be met by lazy people. We are making plans in the hope that one day they will bring those services to us here at the community.” John, age 27, member of a savings and lending group

These two quotes indicate that community group members measure themselves against expectations coming from outside, which in this case is the perceived ‘laziness’ of community members. The quote by Tom underlines how community groups explicitly seek to distance themselves from this representation when NGOs are around in the hope that this will attract a partnership. Similarly, Mercy, a member of the farmers association, when reflecting upon their experiences of working with an NGO, spoke about how recognition of their compliance and agency could lead to further support in the future.

“At the end of the day we get recognition. When the officials of the organisation return to their headquarters, they might include us in some of their future programmes.” Mercy, age 28, member of a farmers’ association.

Earlier we noted that a community group that had received capacity building support from an NGO were staging plays discouraging a so-called ‘laziness’ in the community. This not only suggests that community group members may have begun to assume this sentiment themselves, but indicates that NGOs may be cementing regimes of working that firmly establish the characteristics of who is deserving of a partnership with an NGO and who is not.

## Discussion

We set out to ascertain how community group members view their role in the HIV response, either on their own or in partnership with NGOs. We did this to better understand some of the processes that lay the foundation for much partnership-working in the HIV response and asked the following questions: How do community groups position themselves for partnerships with aid and development agencies? How do representations of self and other shape the foundation of aspiring partnerships? How do power-asymmetries, reflecting the aid architecture, manifest themselves in community groups’ strategic positioning? We will in this discussion react to these questions through a reflection and discussion of key findings.

**Fig. 1:**
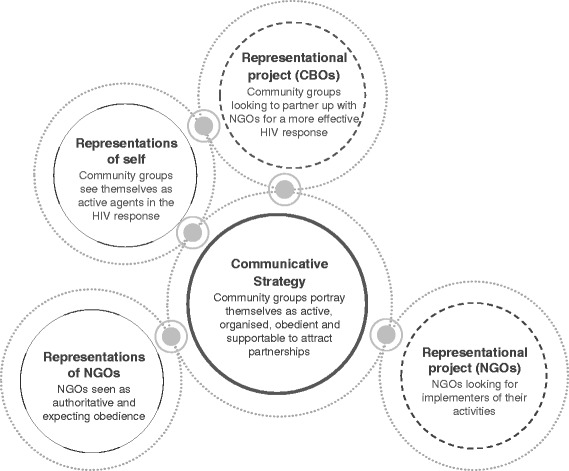
Potential dynamics undermining aspiring efforts for more equitable partnerships from the get-go

We drew on Aveling and Jovchelovitch’s psychosocial theory of partnerships to help us disentangle how instantiations of ‘self’ and ‘other’, as well as encounters with the experience and knowledge of others ([25], p.35) shape community-NGO partnership working. It is clear from our findings that community group members saw themselves, and the community group structure, as important to the HIV response (representations of self). The group setting provided members with an opportunity to help each other during times of hardship, and occasionally, to look beyond the group and assist others in the community. The language they used to explain the benefits of coming together, not only exemplify their agentic capabilities, but is consistent with a broad African ethic of redistribution and reciprocity [30] – key to the HIV response [1, 14, 31, 32]. Accepting there is a limit to what they can do in isolation, community group members actively sought to position themselves for partnerships with more resourceful organisations (representational project). NGOs were perceived as authoritarian, expecting a certain professionalization of the community groups, and whose teleological aim was to identify obedient community groups who could implement their activities (representations of other). Goffman’s [33] dramaturgy provides a useful metaphorical technique to explain how the community groups, in the context of these knowledge encounters, positioned themselves for partnerships. We observed community groups, through ‘impression management’, and based on recognition that NGOs were looking for local partners (representational project), to try and present a ‘supportable’ version of themselves. This involved first some ‘back stage’ work in terms of coming together, and successfully working in formal community group structures, and second, a ‘front stage’ performance, which involved representing themselves as active, organised and obedient actors in the HIV response (communicative strategy). Key to this process was social representations of self and NGOs, ‘the audience’ to whom they were putting on a front, and the role of these representations in shaping perceptions of how best to perform and position themselves as attractive to NGOs for partnership-working (see Fig. 1).

Our findings speak to some deep-seated power asymmetries between local community structures and NGOs. Although the community groups represented themselves as key contributors to the HIV response, such discussions were followed by recognition of their own limits and dependency of external change agents, like NGOs, for a more impactful response. In discussions about partnership-working with external change agents, NGOs were described as authoritarian and prescriptive, entering the communities with pre-packed aid solutions. These observations resonate with a survey distributed to 439 civil-society organisations in six sub-Saharan African countries, which noted that civil-society organisations in the region remain largely involved as service providers for externally formulated programmes, and offered few opportunities to participate in the planning and design of programmes [2].

These processes, coupled with the need for local community structures to strategically position themselves for partnerships with NGOs, highlight just how partnership-workings may be infused with relations of power from the get-go. Looking at a Partnership for Governance Reform initiative in Indonesia, Crawford ([34]: p. 156) found international agencies to exert their power over local actors in driving the partnership agenda forward, resulting in what he calls a “myth of partnership” permeated by an “ongoing exercise of power”. Although our findings resonate with this one-dimensional donor-recipient dichotomy, some commentators have warned against such accounts, arguing they are over simplistic and assume a level of homogeneity at either ‘side’ [35]. While we are in full agreement that stakeholders in partnership-working can “wear multiple hats” (ibid., p. 924), the social representations emerging from the community groups in southern Zimbabwe do suggest instances of ‘us’ and ‘them’.

The social representations identified in this paper form the symbolic field in which meanings and understandings of self and other are constructed [36]. We noted, that in the process of positioning themselves for NGO partnership-working, there is a risk that community groups may internalise some of their perceptions of how NGOs ‘see’ them, for instance ‘lazy’, as articulated by some of our participants. In a worst case scenario such perceptions can come to constitute how they see themselves, and become part of their own development initiatives, such as staging plays to discourage what one of our participants referred to as ‘laziness’. The use of such language, spoken by community members who aspire to, or have had experience of NGO partnership-working, arguably reflects an elitist distancing, more than a reality of some community members being lazy. Escobar ([37]: p.5) refers to such processes, in a development context, as a ‘colonisation of reality’, claiming that “certain representations become dominant and shape indelibly the ways in which reality is imagined and acted upon”. While it is difficult for us, in this study, to disentangle whether the ‘reality’ we report on is indeed imagined, or instances of subversion, with community structures ‘playing the game’ [38], it is clear that the scale of the HIV epidemic, and the institutions responding to HIV, have influenced how communities mobilise support, come together and view themselves and external change agents.

Our findings are constrained by some methodological limitations. First, as with any qualitative study, our findings may not generalise to other settings, as our geographic and cultural environment may well vary from others. Second, the study relies in self-reported data from community group members only. It is difficult for us to ascertain what happens in practice. This however does not negate the fact that the social representations of self (community groups) and other (NGOs) discussed in this paper, provide insight to some of the processes that may lay the foundation for partnership-working.

As we believe NGOs play a critical role in the HIV response, and do not wish to misconstrue the important work of NGOs, our findings should be located within a broader understanding of the AIDS governance system (cf. [39]) in which NGOs operate. This includes recognition of how financial accountability increasingly shapes and constrains the relationships NGOs have with their local partners and their donors. Ann Swidler [38] has argued that increasingly rigid bureaucratic controls and institutional isomorphism limits the opportunity for NGOs to translate and tinker programmes to fit the reality of different actors. Such realities, coupled with the growing difficulty for NGOs to attract long-term and multi-sectorial funding, have led Kelly and Birdsall to conclude that “the funding environment in which they [large-scale HIV programmes] exist actively undermines the unique contributions usually attributed to CSOs and it has done relatively little to strengthen the capacity of the sector as a whole” ([2], p.1587). Cornish and colleagues argue that this ‘new managerialism’ forces NGO workers to conform to rigid global management standards, which are ill-suited to engage and empower local actors [22]. It is arguably this context which has led to the failure of some NGOs in our study setting to engage with community groups in a meaningful and empowering way, resulting in social representations that influence how community groups position themselves for NGO partnership-working.

## Conclusion

Many community groups in Zimbabwe recognise their role in the HIV response and actively navigate representational systems of self and other to showcase themselves as capable actors. While this commitment is admirable, the dynamics that govern this process reflect knowledge encounters and power asymmetries that are conditioned by the aid architecture, undermining aspiring efforts of more equitable partnerships from the get-go. Aveling and Jochelovitch [25] argue that it is “within the scope of development organisations to reflect on how the institutional conditions they sustain support or undermine the renegotiation/re-elaboration of particular representations” (p. 43). Our findings provide plenty of opportunities for such reflection. One, development organisations, NGOs and their donors alike, need to come together and reflect on how their ‘new managerialist’ workings, often centred around short-term ‘upward’ accountability to donors, political leaders, and tax payers, may in fact feed processes that lead to disingenuous partnership workings that contradict their intended goal to empower communities in a way that takes full advantage of their engagement and participation in health programmes. Two, and relatedly, there is a need for greater elasticity in the HIV funding and governance system. NGOs need to have the space and flexibility to be able to act as brokers between incongruent worlds. They need to have the time and resources to be able to involve and incorporate the views of local people at all stages of the programme cycle. Three, this leads us to recommend a complete re-think of accountability altogether. We need to re-balance the focus, and shift the attention more towards our accountability to local people, the intended beneficiaries of a programme [40]. Only by developing accountability mechanisms that take a more long-term, strategic and local view, will we be able to challenge how the community members view themselves and external actors, which may undermine aspiring efforts for more equitable partnerships from the get-go.

## Abbreviations

AIDS: Acquired Immune deficiency syndrome
CBO: Community-based organisation
CSO: Civil-society organisation
FGD: Focus group discussion
HIV: Human immunodeficiency virus
NGO: Non-governmmental organisation
